# Identification and analysis of the interaction network of African swine fever virus D1133L with host proteins

**DOI:** 10.3389/fmicb.2022.1037346

**Published:** 2022-11-03

**Authors:** Yu Hao, Jinke Yang, Bo Yang, Ting Zhang, Xijuan Shi, Xing Yang, Dajun Zhang, Dengshuai Zhao, Wenqian Yan, Lingling Chen, Xiangtao Liu, Haixue Zheng, Keshan Zhang

**Affiliations:** State Key Laboratory of Veterinary Etiological Biology, College of Veterinary Medicine, Lanzhou Veterinary Research Institute, Chinese Academy of Agricultural Sciences, Lanzhou University, Lanzhou, China

**Keywords:** African swine fever virus, D1133L, protein–protein interaction network, VIM, TRIM21, TUFM

## Abstract

African swine fever (ASF) is a contagious and lethal hemorrhagic disease in pigs; its spread results in huge economic losses to the global pig industry. ASF virus (ASFV) is a large double-stranded DNA virus encoding >150 open reading frames. Among them, ASFV-encoded D1133L was predicted to be a helicase but its specific function remains unknown. Since virus-host protein interactions are key to understanding viral protein function, we used co-immunoprecipitation combined with liquid chromatography-mass spectrometry to investigate D1133L. This study describes the interaction network of ASFV D1133L protein in porcine kidney PK-15 cells. Overall, 1,471 host proteins that potentially interact with D1133L are identified. Based on these host proteins, a protein–protein network was constructed. Gene ontology and Kyoto Encyclopedia of Genes and Genomes enrichment analyses showed that cellular D1133L-interacted proteins are involved in the ribosome, spliceosome, RNA transport, oxidative phosphorylation, proteasome, and DNA replication. Vimentin (VIM), tripartite motif-containing protein 21 (TRIM21), and Tu translation elongation factor (TUFM) were confirmed to interact with D1133L *in vitro*. VIM or TRIM21 overexpression significantly promoted ASFV replication, but TUFM overexpression significantly inhibited ASFV replication. These results help elucidate the specific functions of D1133L and the potential mechanisms underlying ASFV replication.

## Introduction

African swine fever (ASF) is a highly contagious and seriously lethal hemorrhagic disease in domestic pigs and wild boars (Gallardo et al., 2015). ASF was first reported in Africa in the early twentieth century, gradually spreading across Europe and Asia in the following century (Gogin et al., 2013; Mushagalusa et al., 2021). In 2018, ASF broke out in China, whose porcine production accounts for half of the global production, spreading rapidly throughout the country within a year (Zhou et al., 2018). ASF caused great economic losses to the global pig industry posing a serious threat to pork safety and supply. Despite researchers’ efforts, there are no safe and efficient ASF vaccines or treatments available, and ASF prevention only relies on the rapid culling of susceptible animals and strict epidemic control measures (Urbano and Ferreira, 2022).

ASF virus (ASFV) is the only member of the *Asfaviridae family* and contains a large linear double-stranded DNA genome. Its genome length ranges between 170 and 194 kbp and encodes >150 proteins (Wang N. et al., 2019; Gaudreault et al., 2020). These viral proteins ensure the ASFV virion life cycle, including invasion, replication and transcription, and host immunomodulation (Wang et al., 2021b). The ASFV DNA polymerase encoded by *G1211R* and *O174L* (Rodriguez et al., 1993; Jezewska et al., 2011), a DNA ligase encoded by *NP419L* (Lamarche et al., 2005), a topoisomerase II P1192R (Coelho et al., 2015, 2016), and a dUTPase E165R (Zhang et al., 2021) are responsible for viral replication. RNA helicases Q706L and QP509L (Freitas et al., 2019), *C315R* encoded TFIIB-like factor (Cackett et al., 2020), and transcription factor SII encoded by *I243L* (Rodriguez et al., 1996) are important for viral transcription and translation. ASFV pB602L, a molecular chaperone protein, facilitates the correct folding and assembly of the viral structural protein B646L (Epifano et al., 2006). And A179L, A224L, EP153R, and pS273R are involved in the regulation of host programmed cell death (Hurtado et al., 2004; Hernaez et al., 2013; Zhao et al., 2022). Other multigene families (MGF) proteins antagonize host innate immunity to further promote virus replication. For instance, MGF360-11 L and MGF505-11R negatively regulate cGAS-STING to attenuate IFN-I expression (Rothan et al., 2019; Yang et al., 2022), and MGF360-9 L inhibits the JAK/STAT pathway and expression of interferon-induced antiviral factors by degrading STAT1 (Zhang K. S. et al., 2022).

Despite increasing research achievement, given ASFV complexity, the function of many ASFV proteins remains unclear. ASFV-encoded D1133L is an intermediate-late protein with nuclear and cytoplasm localization during infection (Hou et al., 2021). ASFV-encoded D1133L contains putative DEXD/H-box motifs characterized by the superfamily II (SFII) and is therefore predicted to be a helicase, and may be involved in ASFV transcriptional initiation (Yáñez et al., 1993). ASFV virions exhibit higher replication efficiency in MA-104 cells overexpressing D1133L, indicating that D1133L has a positive effect on ASFV replication (Zhang T. et al., 2022). However, D1133L’s exact functions during ASFV infection have not been further explored. Considering that the interaction between virus-host is critical for viruses to regulate host cell function and ensure virus efficient replication, revealing the interactions between D1133L and host proteins may help elucidate D1133L function (Weitzman and Fradet-Turcotte, 2018; Strumillo et al., 2021). As a high-throughput screening method, the combination of co-immunoprecipitation (co-IP) and liquid chromatography-mass spectrometry (LC–MS) has been widely used to study the interaction between virus and host proteins, such as the H5N1 influenza A virus (Wang Q. et al., 2019), Pseudorabies Virus (Rothan et al., 2019), and ASFV (Yang et al., 2021). Therefore, we extended the approach to D1133L and further explored its significance by identifying and analyzing D1133L-interacted host proteins.

Through Co-IP and LC–MS, we finally identified 1,471 proteins that possibly interact with D1133L in PK-15 cells. Based on these host proteins, a protein–protein interaction network was constructed, and bioinformatics enrichment analysis was performed. Three host proteins associated with viral infections, vimentin (VIM), tripartite motif-containing protein 21 (TRIM21), and Tu translation elongation factor (TUFM) interacted with D1133L *in vitro*. Subsequently, the role of VIM, TRIM21 and TUFM on ASFV replication was investigated *in vitro*. Ectopic VIM and TRIM21 expression promoted ASFV replication but TUFM hindered ASFV replication in MA-104 cells. These results can serve as the basis for further exploration of the explicit role of D1133L in regulating ASFV replication and cellular activity.

## Materials and methods

### Cells and viruses

According to the previously described bronchoalveolar lavage method (Carrascosa et al., 1982), Primary porcine alveolar macrophages (PAMs) were prepared and cultured in Roswell Park Memorial Institute medium (Gibco) containing 10% porcine serum and maintained at 37°C, 5% CO_2_. Porcine Kidney 15 (PK-15) and microbiological associates-104 (MA-104) cells were cultured in Dulbecco’s modified Eagle medium (Gibco) with 10% fetal bovine serum (FBS, Gibco), 100 μg/mL streptomycin, and 100 U/mL penicillin and maintained at 37°C, 5% CO_2_. MA-104 cells, a commercial cell line, was purchased from China Center for Type Culture Collection (GDC0041, Wuhan, China).

The CN/GS/2018 ASFV strain, genotype II ASFV, was isolated in Lanzhou Veterinary Research Institute (Lanzhou, China) and stored at −80°C (Zhang K. S. et al., 2022).

### Plasmid and antibodies

Plasmids encoding porcine VIM (Gene ID: 100522394), TRIM21 (Gene ID: 100302538), and TUFM (Gene ID: 100516488) were constructed by inserting the synthesized sequence into pCDNA3.1 with Myc tags fused to the 3′ end and performed at Wuhan GeneCreate Biological Engineering (Wuhan, China).

The preparation work of the anti-D1133L mouse monoclonal antibody was carried out by Wuhan GeneCreate Biological Engineering (Wuhan, China). Anti-Myc rabbit monoclonal antibody (2276S), Alexa Fluor 488 anti-rabbit IgG (4416S), and Alexa Fluor 594 anti-mouse IgG (8890S) were purchased from Cell Signaling Technology (CST). HRP-conjugated goat anti-mouse IgG LCS antibody (A25012) was purchased from Abbkine and used to alleviate heavy chain interference. HRP-conjugated Affinipure Goat Anti-Mouse IgG (H&L) (SA00001-1) and HRP-conjugated Affinipure Goat Anti-Rabbit IgG (H&L) (SA00001-2) were purchased from Proteintech (Wuhan, China).

### Cell transfection

To transfect the related plasmid, MA-104 cells were inoculated onto 12-well cell culture plates or 10 cm cell culture dishes and grown till reaching about 80% confluence after transfection. Polyplus jetPRIME (PT-114-15) transfection reagents were used. Each well of the 12-well plate and each 10 cm cell culture dish were transfected with 3 or 10 μg related plasmid, respectively. After 24 h, the transfected plasmid was successfully expressed and the following experiment, i.e., viral infection, was performed.

### Liquid chromatography mass spectrometry

The experiments were performed on a Q Exactive mass spectrometer coupled with an Easy nLC (Thermo Fisher Scientific). The peptide mixture was loaded onto the reversed-phase column packed in-house in buffer A (0.1% formic acid in HPLC-grade water) and separated with a linear gradient of buffer B (0.1% formic acid in 84% acetonitrile). The flow rate was controlled to 300 nl/min, the total operation was 60 min. MS data were acquired using a data-dependent top 10 method, dynamically choosing the most abundant precursor ions from the survey scan (300–1800 m/z) for HCD fragmentation. To determine if the target value was based on predictive automatic gain control, the dynamic exclusion duration was set to 20 s. Survey scans were acquired at a resolution of 70,000 at m/z 200, and the resolution for HCD spectra was set to 17,500 at m/z 200. The normalized collision energy was 27 eV, and the underfill ratio, which specifies the minimum percentage the target value is likely to reach at maximum fill time, was defined as 0.1%. The instrument was run in peptide recognition mode.

MS/MS spectra were searched using the MASCOT engine (Matrix Science, London, United Kingdom; v.2.2) against the UniProt Galagidae protein database. Relevant details of the protein identification process are as follows: 20 ppm peptide mass tolerance, 0.1 Da MS/MS tolerance, two missed cleavage, fixed modification = carbamidomethyl (C), variable modification = oxidation (M), ion score >20, and FDR <0.01 at peptide and protein levels. Non-specific interactions were removed by eliminating the detected proteins in the negative control sample.

### Construction and analysis of PPI network

Cytoscape v.3.7.1. was used to construct the D1133L-host protein interaction network based on all obtained data, and the STRING database was used to establish the host protein–protein interaction (PPI) network. Topological parameters and central measures of the network were calculated using a network analyzer tool in Cytoscape v.3.7.1.

### Protein functional enrichment analysis

Gene ontology (GO) enrichment analysis was performed using Cytoscape v.3.7.1, selected over-representation analysis (ORA) as the analysis strategy with the value of *p* <0.05. The Kyoto Encyclopedia of Genes and Genomes (KEGG) database was accessed using the KOBAS software *via* hypergeometric test, with a corrected value of *p* <0.05.

### Co-immunoprecipitation

Cells were collected and lysed using NP-40 lysis buffer containing PMSF for 30 min at 4°C. An ultrasonic instrument was used to further lyse the cells over a total of 2.0 min, 20 kHz frequency, and 25 W sonicator power. The treated cell lysate was incubated for 18 h at 4°C with the specified antibodies or the corresponding species IgG as controls. Then, protein A/G agarose beads (Roche) were mixed for 3 h with cell lysate to bind antibodies in it. Ultimately, the beads were collected by centrifugation and washed with NP-40 lysis buffer. Sodium dodecyl sulfate (SDS) loading buffer was mixed with the beads and boiled to conduct the next step.

### Immunoblotting analyses

For Western blotting, the whole proteins were separated using 10% SDS-polyacrylamide gel electrophoresis (80 V, 30 min; 120 V, 60 min) and then migrated to the nitrocellulose (NC) membrane (100 V, 90 min). Then, NC membranes containing proteins were blocked with skim milk of 5% for 1 h. After washing three times with Tris-buffered saline with 0.1% Tween 20 (TBST) for 10 min each, the NC membranes were incubated with specific antibodies at 4°C overnight. The next day the NC membranes were washed again with TBST (3×, 10 min each) and were incubated with the appropriate HRP-conjugated IgG secondary antibody for 2 h at room temperature. Finally, an electrochemiluminescence solution was used to react with HRP on NC membranes, and images were eventually acquired using the Odyssey infrared imaging system.

### Indirect immunofluorescence assay

MA-104 cells were incubated and treated in dedicated cell confocal imaging dishes. The cells were fixed with 4% paraformaldehyde for 30 min, permeabilized with 0.2% TritonX-100 for 10 min, and blocked in 5% BSA for 1 h. Next, cells were incubated with corresponding antibodies for 12 h at 4°C. Then, it was incubated with Alexa Fluor 488 anti-rabbit IgG and Alexa Fluor 594 anti-mouse lgG for 2 h, and stained with 4-methyl-6-phenylindole for 10 min. The samples were imaged by the Leica SP2 confocal system (Leica Microsystems, Wetzlar, Germany).

### Real-time quantitative PCR

Total RNA was extracted from MA-104 cells using the TRIzol reagent (Thermo Fisher Scientific) and was reverse transcribed using the PrimeScript RT kit (TaKaRa). qPCR was performed using the PowerUp SYBR Green Master Mix on the ABI StepOnePlus system. All data were analyzed using the StepOnePlus software, and the relative mRNA level of genes was normalized based on the GAPDH mRNA level. At last, the relative expression level of mRNA was calculated based on the comparative cycle threshold (2^-ΔΔCT^) method. ASFV P72 primer sequences used in this study: ASFV-P72-F: 5’-TGC GAT GAT GAT TAC CTT-3′; ASFV-P72-R: 5′-ATT CTC TTG CTC TGG ATA C-3′; GAPDH-F: 5′-GAG TCA ACG GAT TTG GTC GT-3′; GAPDH-R: 5′-GAC AAG CTT CCC GTT CTC AG-3′.

### Viral titration (50% hemadsorption doses)

The anticoagulated whole blood collected from healthy pigs was washed three to five times with sterilized PBS (0.1 M, pH 7.2) containing 1% penicillin–streptomycin and centrifuged at 350× g for 3 min each time. Porcine red blood cells (RBCs) were obtained when the supernatant of porcine anticoagulant whole blood is close to colorless and transparent. PAMs were incubated in 96-well plates and 30 μl of 1% porcine RBCs were added to each well. Virus samples were diluted to 10^−1^, 10^−2^, 10^−3^, 10^−4^, 10^−5^, 10^−6^, and 10^−7^ and added into the 96-well plate containing PAMs and porcine RBCs at 0.1 mL per well. Eight repeat wells were set for each sample dilution. The adsorption of RBCs was observed for a week. Fifty percent hemadsorption doses (HAD_50_) were calculated according to the Reed-Muench method (Biacchesi et al., 2005).

### Statistical analysis

The experimental results were analyzed using GraphPad Prism v.8.0 (San Diego, CA, United States). All data are presented as means ± standard deviations (SDs) from three independent experiments. *p < 0.05 was considered statistically significant. **p < 0.01 and ***p < 0.001 was considered highly statistically significant, ns means no difference.

## Results

### Identifying ASFV D1133L-interacting factors in PK-15 cells by co-IP and LC–MS

**Figure 1:**
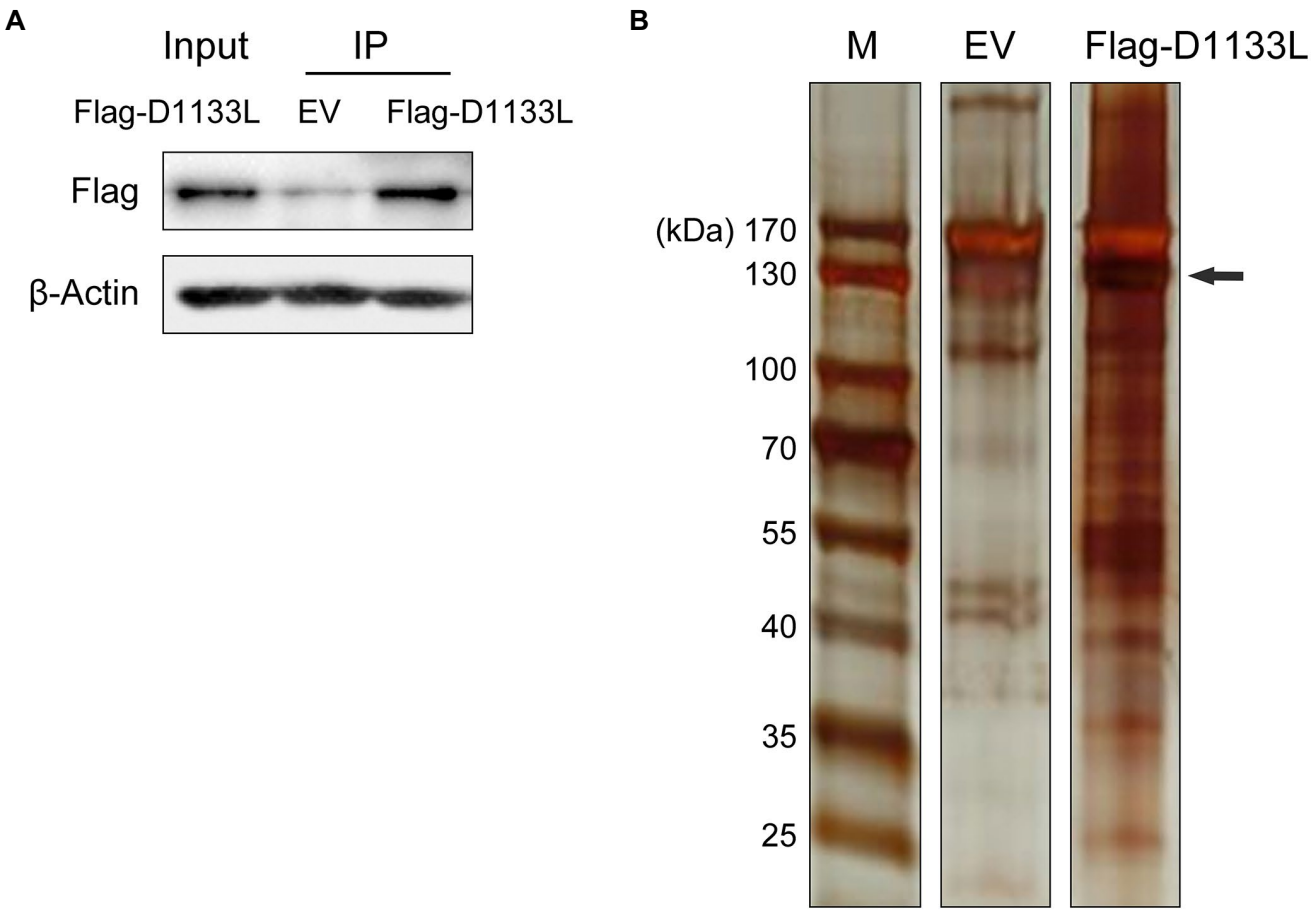
Western blotting to confirm the expression of exogenous Flag-D1133L in PK-15 cells and silver staining to show the enrichment for host proteins interacting with D1133L. PK-15 cells were transfected with an empty vector (10 μg) or Flag-D1133L (10 μg), collected, and lysed 24 h post-transfection. **(A)** Western blotting detected Flag-D1133L in whole-cell lysates. **(B)** Co-IP was performed on cell lysates with the anti-Flag antibody. IP-treated samples were detected through Western blotting and silver staining. Lane 1, marker; Lane 2, empty Flag vector (EV) as control; Lane 3, Flag-D1133L enriched protein products; Flag-D1133L is indicated by the black arrow. Data were tested three times independently.

To explore the potential host proteins that interact with D1133L, a 3 × FLAG tag D1133L plasmid or empty FLAG plasmid was transfected into PK-15 cells for Co-IP and LC–MS analysis (Figure 1A). D1133L and D1133L-interacting host factors were immunoprecipitated by using an anti-Flag antibody. First, Flag-D1133L plasmid was successfully expressed in PK-15 cells t and was immunoprecipitated successfully and specifically (Figure 1A). Compared to the empty FLAG control, the silver stain showed clear Flag-D1133L at the expected molecular weight (130 kDa) and its interaction partners, indicating the specific enrichment of D1133L-associated factors (Figure 1B).

Then, LC–MS identified the D1133L-interacting proteins in PK-15 cells. The nonspecific background binding data in the D1133L binding protein data were subsequently eliminated by comparison with an empty FLAG control. The remaining interactions were analyzed by significance analysis of the interactome. Finally, 1,471 cellular proteins were found to interact with D1133L (Supplementary Table S1).

### ASFV D1133L-host proteins interactome

**Figure 2:**
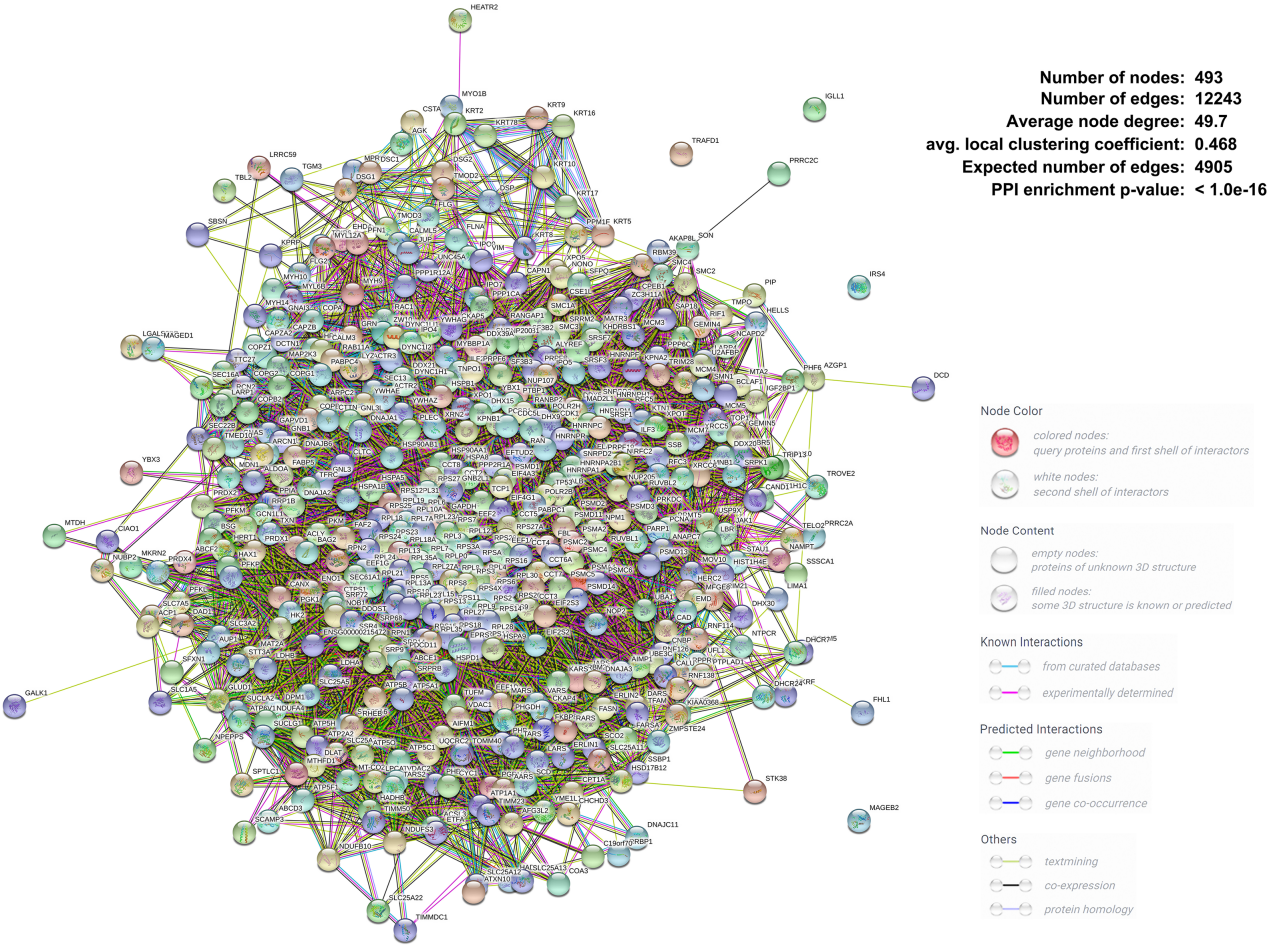
Construction and analysis of the virus-host protein–protein interaction network. The map of ASFV D1133L-interacting host proteins was constructed and plotted using the network analyzer tool, Cytoscape v.3.7.1. Each node represents a protein, and the corresponding NCBI abbreviation is annotated next to it. Detailed PPI statistics are listed in the upper right corner of the graph. Differently colored inter-protein links indicated different PPI patterns; the specific meaning is shown in the note in the lower right corner of the Figure.

Proteins are cellular functional performers so PPI identification is indispensable for molecular biology. The interaction of viral proteins and host proteins is essential for viral replication in cells. Accordingly, a PPI network between ASFV D1133L-interacting host proteins was constructed and protein interactions were comprehensively analyzed through the STRING database (Figure 2). The observed number of edges (12243) for the PPI network was significantly higher than the expected number of edges (4905) for the given number of nodes (493), implying that the host proteins from our data exist more interactions than expected for a random group of proteins. Such enrichment manifested that the D1133l-interacting host proteins are partially clustered as the multi-protein complex affecting D1133L function.

### Go enrichment analysis

**Figure 3:**
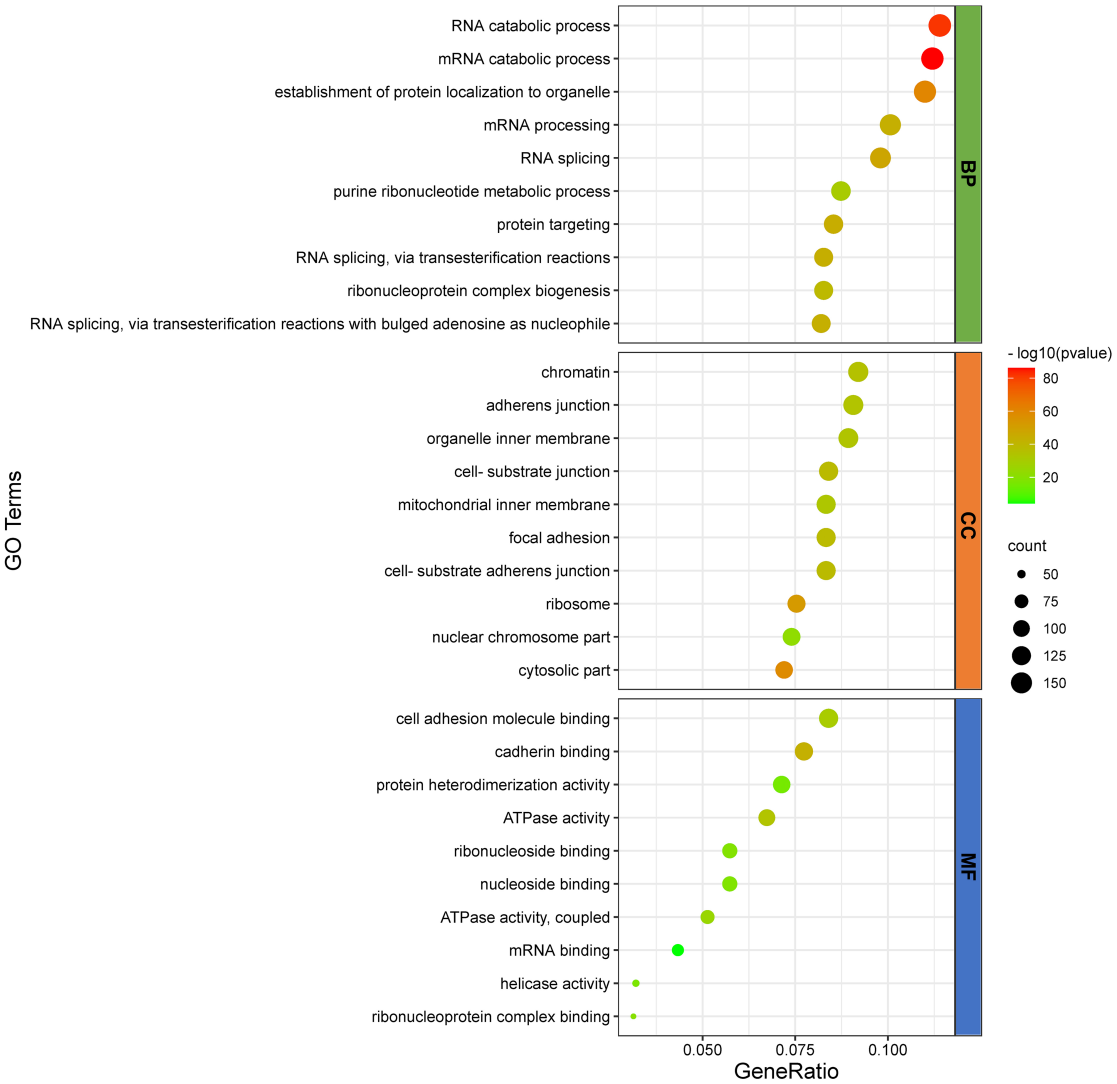
GO functional classification of D1133L-interacting host proteins. The distributions are summarized in three main categories: biological process (Fagerberg et al., 2014), and cellular component (CC), molecular function (MF). The *X*-axis indicates the gene ratio and the *Y*-axis indicates the GO terms, value of *p* <0.05.

To further infer the main biological functions of the host proteins interacting with D1133L, GO analysis was performed (Figure 3). Over-representation analysis (ORA) was chosen as a better analysis strategy to obtain more biologically significant results. GO analysis indicated that these proteins are mostly involved in RNA and mRNA catabolic processes, the establishment of protein localization in organelles, mRNA processing, RNA splicing, and purine ribonucleotide metabolic processes were enriched under the biological process category; chromatin, adherens junction, cell-substrate junction, ribosome, nuclear chromosome part were enriched under the cell component category; and cell adhesion molecule binding, cadherin binding, ATP activity, ribonucleoside binding, mRNA binding, and helicase activity were enriched under the molecular function category. Collectively, the GO annotation and analysis of all target proteins inferred that D1133L might participate in RNA metabolism, DNA replication, and ribosomal function.

### KEGG pathway enrichment analysis

**Figure 4:**
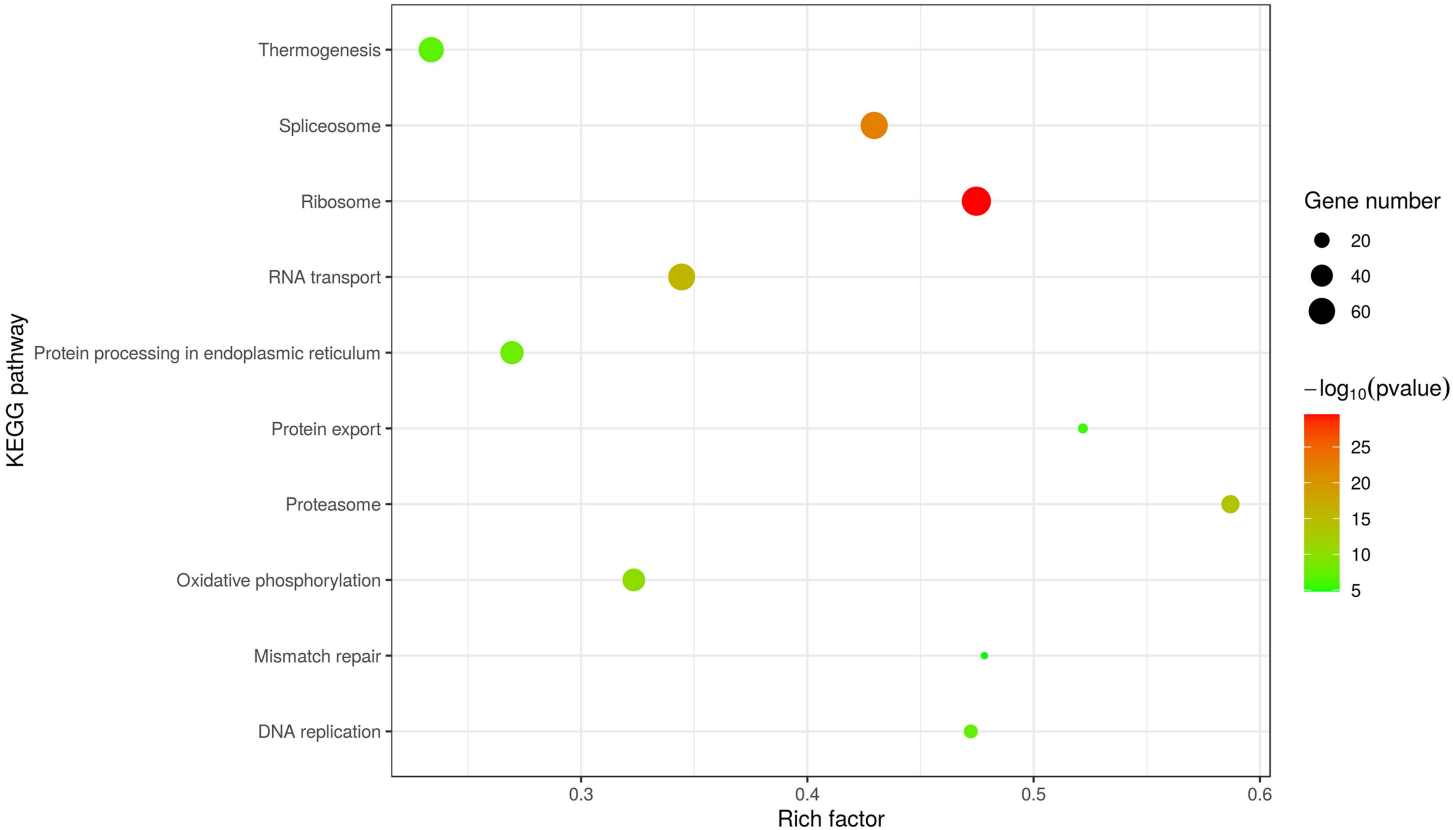
Scatter plot of D1133L interacting host proteins enriched KEGG pathways statistics. The top 10 enriched pathways at *p* < 0.05 are shown.

In addition, KEGG analysis was performed to further understand and predict the cellular pathways of metabolism and signal transduction involved in D1133L-interacting proteins (Figure 4). Interestingly, the majority of D1133L-interacting host proteins were closely related to the ribosome, spliceosome, RNA transport, and oxidative phosphorylation pathway; and a small number of D1133L-interacting factors appeared in the proteasome, DNA replication, and protein export pathway, suggesting the potential roles of these proteins in ASFV infection.

### Validating the interaction between LC–MS-identified host proteins with ASFV D1133L

**Figure 5:**
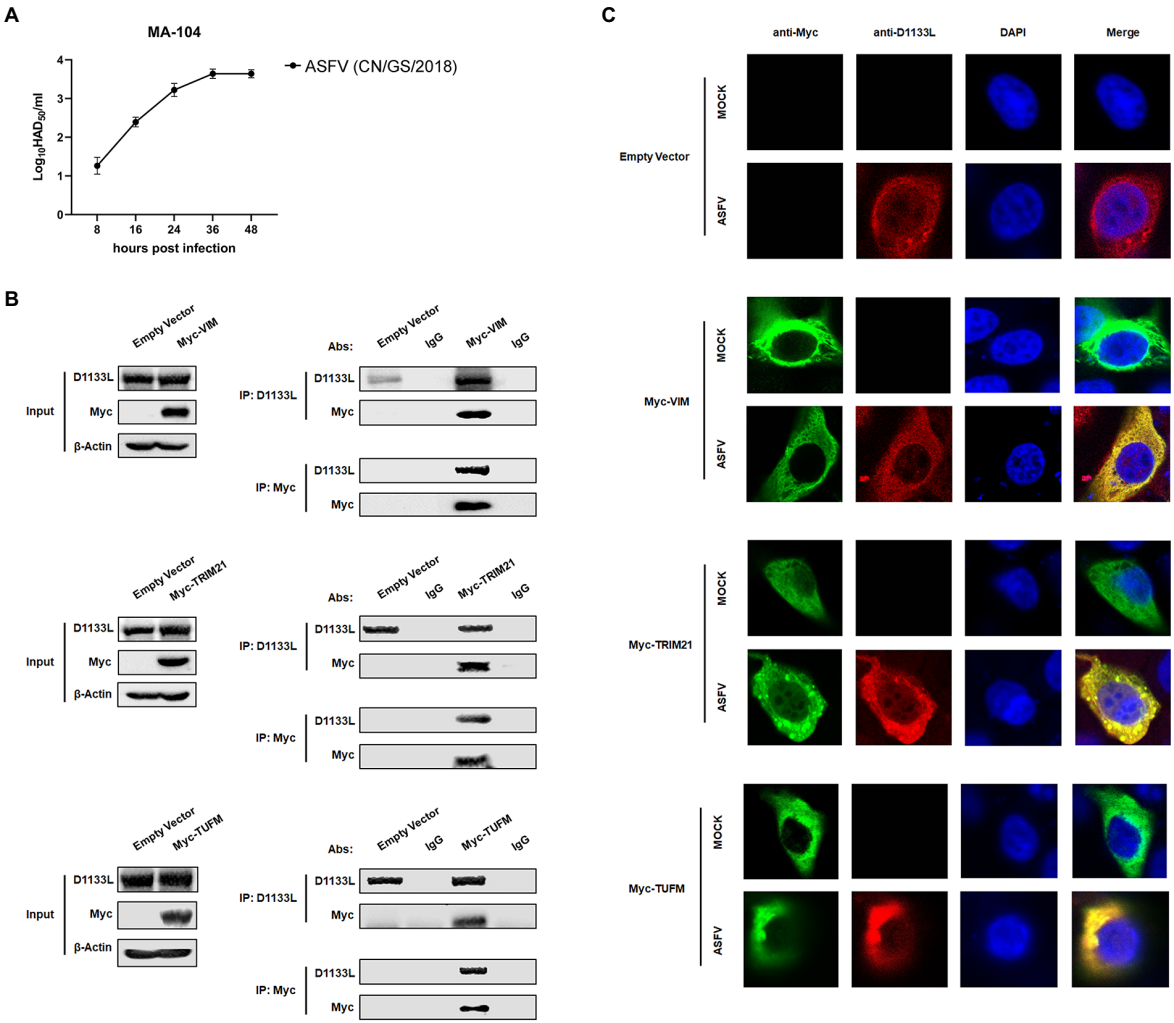
Verification of the interaction between ASFV D1133L and host proteins VIM, TRIM21, and TUFM. **(A)** MA-104 cells were infected with ASFV (1.0 MOI), and the viral titer was determined at 8, 16, 24, 36, and 48 hpi by HAD_50_ methods. **(B)** MA-104 cells were transferred into a 10 cm culture dish, and the empty vector plasmid, Myc-VIM, Myc-TRIM21, or Myc-TUFM was transfected (12 μg/dish) into the cells at 80% confluence. At 24 h post-transfection, MA-104 cells were infected with ASFV (1.0 MOI). After 24 h, the anti-D1133L antibody or anti-Myc antibody was added to the cell lysates for IP or reverse IP. IgG was used as a control. After SDS-PAGE separation and western blotting, proteins were detected by corresponding antibodies. Beta-actin was selected as the internal loading control. **(C)** MA-104 cells were plated into 20 mm culture dishes, and empty vector plasmid, Myc-VIM, Myc-TRIM21, or Myc-TUFM were transfected (3.5 μg/dish) into the cells at 80% confluence. Transfected MA-104 cells were infected with ASFV (1.0 MOI). At 24 h post-infection (hpi), cells were treated according to the indirect immunofluorescence step and observed through the Leica laser confocal microscope.

To further validate the interaction between LC–MS identifying host proteins and D1133L, we performed Co-IP and reverse Co-IP experiments *in vitro*. VIM, TRIM21, and TUFM were selected from the protein library of D1133L interactions, as all of them are associated with various viral replication. Considering that MA-104 cells had recently been shown to be infected with ASFV and the inefficiency of transfection at PAMs (Rai et al., 2020), we performed ectopic VIM, TRIM21, and TUFM expression in MA-104 cells to assess their interaction with D1133L. First, the proliferation kinetics of ASFV in MA-104 cells showed that the viral titer increased rapidly within 24 h and are maximal at 36 h after the infection (Figure 5A). After that, the results of Co-IP and reverse Co-IP all indicated that D1133L does accurately interact with VIM, TRIM21, and TUFM (Figure 5B).

Next, an indirect immunofluorescence assay was performed to investigate co-localization between D1133L and VIM, TRIM21, and TUFM. MA-104 cells were transfected with Myc-VIM, Myc-TRIM21, and Myc-TUFM or empty vector plasmid as control. The transfected cells were divided into two groups: the uninfected mock group and the infected group (1.0 MOI of ASFV). The imaging results showed that D1133L co-localized with VIM, TRIM21, and TUFM in infected cells (Figure 5C).

Taken together, these data indicated that ASFV D1133L interacted with VIM, TRIM21, and TUFM *in vitro* and validated the data generated from the LC–MS-based proteomic analysis.

### VIM and TRIM21 overexpression significantly promoted and TUFM overexpression significantly inhibited ASFV replication in MA-104 cells

**Figure 6:**
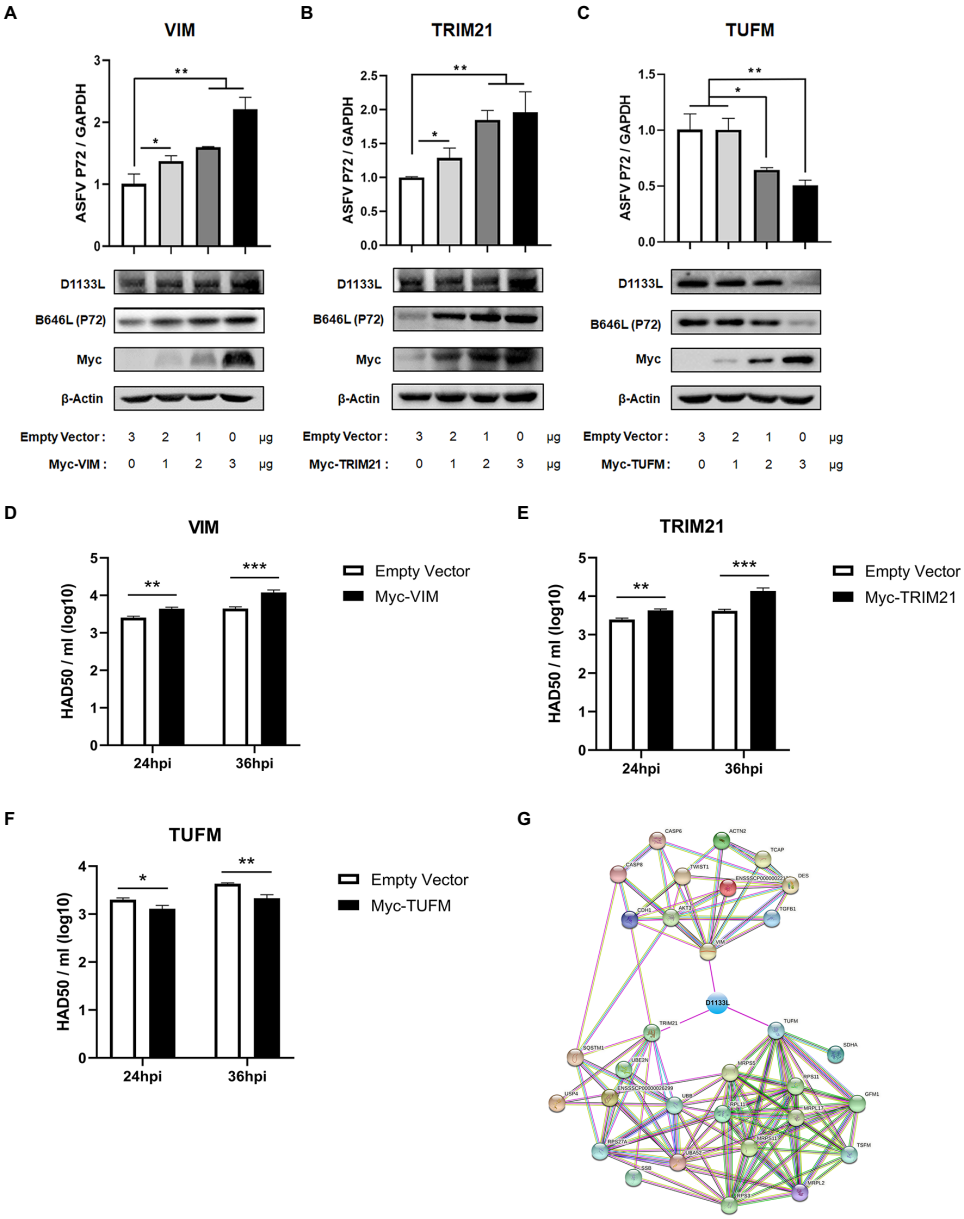
ASFV D1133L interacting host proteins, VIM, and TRIM21 enhanced the replication of ASFV, but TUFM inhibited ASFV replication. **(A–C)** MA-104 cells were cultured on 12-well plates. When the confluence reached 80%, MA-104 cells were transfected with the empty vector, Myc-VIM, Myc-TRIM21, or Myc-TUFM in a dose-dependent manner. At 24 h after transfection, 1.0 MOI dose of ASFV infected the MA-104 cells. Cell samples were collected at 24 h postinfection, and the expression level of the B646L gene (P72) and D1133L protein was detected through RT-qPCR and Western blotting. GAPDH and β-actin were used as internal reference controls. **(D–F)** When the MA-104 cells were laid on 12-well plates and the confluence degree reached 80%, empty vector (3 μg/well), Myc-VIM (3 μg/well), Myc-TRIM21 (3 μg/well), or Myc-TUFM (3 μg/well) were transfected into the cells. At 24 h after transfection, 1.0 MOI dose of ASFV was added to the cell. The titers of ASFV were detected by HAD_50_ at 24 and 36 hpi. **(G)** ASFV D1133L binding VIM, TRIM21, and TUFM-cellular protein interaction network. The map was established by the STRING Protein–Protein Interaction Networks Functional Enrichment Analysis Database (https://www.string-db.org/). Data were tested three times independently. *p<0.05, **p<0.01, ***p<0.001.

VIM, TRIM21, and TUFM were experimentally identified as host interacting factors of ASFV D1133L but their effect on ASFV replication is unclear. Thus, to further determine the influence of VIM, TRIM21, and TUFM on ASFV replication, MA-104 cells were separately transfected with plasmids encoding Myc-VIM, Myc-TRIM21, Myc-TUFM at three transfection doses: 1 μg, 2 μg, and 3 μg. Empty Myc-pcDNA 3.1(+) vector was used as control. At 24 hpi, ASFV infected MA-104 cells at 1.0 MOI dose. RT-qPCR results showed that at 24 h post-infection, ASFV *B646L* gene (encoding ASFV P72 structural protein) expression gradually increased with increasing VIM or TRIM21 transfection dose (Figure 6A-B), but gradually decreased with TUFM (Figure 6C). Western blotting showed that, compared to controls, inhibits D1133L and P72 in a dose-dependent manner (Figure 6A-C). HAD_50_ assay also proves this point. At 24 and 36 hpi, the viral titer of samples overexpressing VIM or TRIM21 were significantly upregulated (Figure 6D-E) but the viral titer of samples overexpressing TUFM were significantly downregulated (Figure 6F). The above results confirmed that the D1133L interacting host proteins VIM and TRIM21 facilitate ASFV replication, whereas TUFM inhibited ASFV replication.

In addition, we constructed another interactions network of cellular partners interacting with VIM, TRIM21, and TUFM using Cytoscape 3.7.1 software (Figure 6G), which might help investigate the potential significance of D1133L related to VIM, TRIM21, or TUFM during the replication lifecycle as well as the pathogenesis of ASFV.

## Discussion

African swine fever, an infectious disease with hemorrhagic characteristics and high mortality rates in domestic porcine and wild boars, has been widely circulated worldwide in the past few decades and caused huge losses to the global pig industry (Galindo and Alonso, 2017). Because of the absence of effective commercial vaccines or drugs, the control of ASF can only rely on strict prevention and rapidly culling of infected pigs (Urbano and Ferreira, 2022). As a large double-stranded DNA virus, ASFV encodes more than 150 open reading frames (ORFs) (Yutin and Koonin, 2012). Despite a lot of effort to explore the ASFV proteins’ function, the molecular mechanism of ASFV replication and pathogenesis, as well as its dependency on host factors are still poorly known.

ASFV replication needs host cellular functions. Virus-host protein interaction is the main way in which ASFV utilizes the host cellular systems, participates in host cellular biological processes as well as interferes with host immunity. ASFV DP71L and I14L, which share sequence similarity with the herpes simplex virus ICP34.5 protein, interact with protein phosphatase 1 (PP1) to dephosphorylate eIF2alpha and avoid PKR-mediated protein synthesis shutdown (Goatley et al., 1999; Rivera et al., 2007; Zhang et al., 2010). Similarly, ASFV mRNA translation initiation depends on eIF4F complex-driven viral mRNA capping (Castello et al., 2009). Protein–protein interaction is also the most usual approach for host anti-viral immunity. Host factor FoxJ1 inhibits ASFV replication by degrading ASFV MGF505-2R and E165R through the autophagy pathway (Ma et al., 2022). Past studies had suggested that ASFV D1133L belongs to the SFII family, and possesses a similar NTP-binding motif and DEXD/H motif (Yáñez et al., 1993). D1133L had therefore been inferred to be a helicase for ASFV, but no further experiments against D1133L confirm these functions. This study uses co-IP and LC/MS to identify the host protein group interacting with ASFV D1133L. We screened and confirmed 1,471 host proteins that may interact with D1133L, and presented them as a PPI network map. The map showed 493 nodes, 4,905 expected edges, and 12,243 observed edges. Compared to expected edges, the significantly more observed edges indicated that there are more interactions than expected. Also, such a PPI map implied that some proteins from our data are clustered as a multiprotein complex relating to replication or transcription. Subsequent protein functional enrichment analysis better elucidated the functions of these interacting proteins of ASFV D1133L. Molecular function, including RNA/mRNA catabolic process, mRNA processing, RNA splicing, and ribonucleoprotein complex biogenesis; cellular function, including chromatin, ribosome, mitochondrial inner membrane, and nuclear chromosome part; biological processes, including ATPase activity, mRNA binding, helicase activity, cadherin binding, protein heterodimerization activity, and ribonucleoside binding was enriched based on GO analysis (Figure 3). The enrichment strongly supports the previously predicted helicase and ATPase activity of D1133L (Baylis et al., 1993; Roberts et al., 1993; Yáñez et al., 1993). In addition, KEGG pathway analysis showed that the spliceosome, ribosome, RNA transport, DNA replication, and proteasome pathway were notably enriched in D1133L-host protein interactions (Figure 4). These cellular processes are important during ASFV infection and hence need special attention during further studies. For instance, spliceosome proteins help the production of the right viral RNA conformation, and ribonucleoproteins assist with viral RNA stability and transport (Will and Luhrmann, 2011; Gilman et al., 2017). Vaccinia virus protein NPH-II, a viral RNA helicase of the DExH family, is involved in RNA unwinding, synthesis of early messenger RNA, and remodeling of RNA-protein complexes (Gross and Shuman, 1996, 1998; Jankowsky et al., 2000; Fairman-Williams and Jankowsky, 2012). Moreover, the other two ASFV RNA helicases of the SFII family, Q706L and QP509L, were noted to have non-redundant functions on ASFV replication (Freitas et al., 2019). And the proteasome system is vital in ASFV proteins-mediated innate immune escape (Riera et al., 2021). Therefore, it is inferred that ASFV D1133L is involved not only in transcription initiation but also in the viral genome replication, viral RNA, and protein metabolism indispensable for ASFV.

We also validated the binary interactions and co-localizes of ASFV D1133L with selected host proteins such as VIM, TRIM21, and TUFM *in vitro* by coimmunoprecipitation and indirect immunofluorescence assays (Figure 5B-C). In addition, the weak distribution of D1133L within the nucleus in ASFV infected MA-104 cells can be observed (Figure 5C). While ASFV replicates in viral cytoplasmic factories, the presence of partial ASFV genome within the host cell nucleus has been previously confirmed (Garcia-Beato et al., 1992; Rojo et al., 1999; Simoes et al., 2015). Thus, intranuclear D1133L may be critical for early replication or transcription of ASFV within the nucleus. Both VIM and TRIM21 are closely related to ASFV infection. VIM is an intermediate filament protein and is important to maintaining cellular integrity (Ridge et al., 2022). ASFV infection induces phosphorylation, rearrangement, and collapse of VIM into characteristic cages that package virus factory and may facilitate ASFV replication in the same way that it facilitates porcine reproductive and respiratory syndrome virus (PRRSV) replication (Heath et al., 2001; Stefanovic et al., 2005; Zheng et al., 2021). E3 ubiquitin ligase TRIM21 exhibits different effects on different viruses. For example, on the one side, TRIM21 restricts porcine epidemic diarrhea virus (PEDV) proliferation by degrading the viral nucleocapsid protein (Wang H. et al., 2021). On the other side, TRIM21 is important for ASFV MGF360-14 L-mediated IRF3 degradation and inhibition of IFN-I production to encourage ASFV replication (Wang et al., 2021a). In addition, TUFM is a mitochondrial outer membrane protein and efficiently binds to the PB2_627E_ of Avian Influenza A Virus inducing mitophagy to limit virus proliferation in human cells (Kuo et al., 2017). But the role of TUFM on ASFV replication is unclear. Next, ectopic VIM, TRIM21, and TUFM expressions were performed in MA-104 cells to assess their impact on ASFV replication. Overexpressing VIM and TRIM21 encouraged ASFV replication, but overexpressing TUFM inhibited it (Figure 6A-F). Such overexpression results are consistent with previous results that VIM and TRIM21 are beneficial to ASFV replication, and also point out for the first time that TUFM acts as a host restriction factor for ASFV replication. Furthermore, the molecular mechanism of how the interaction of D1133L with VIM, TRIM21, and TUFM affects ASFV replication needs to be further studied, and that will be useful for better understating the function of D1133L and host proteins in ASFV replication and pathogenesis.

In conclusion, the interaction between ASFV D1133L and cellular proteins was systematically screened in PK-15 cells transfected with D1133L. Based on 1,471 potential D1133L-interacted host proteins, a PPI network was constructed, and their potential functions were investigated by GO and KEGG enrichment analyses. The results of enrichment analyses supported past predictions of D1133L being a helicase with ATPase activity and further inferred its importance in the regulation of viral RNA metabolism. Moreover, three randomly selected proteins associated with viral infections: VIM, TRIM21, and TUFM were confirmed to interact with D1133L by co-IP and IFA assays. VIM and TRIM21 overexpression in MA-104 cells increased ASFV replication, whereas TUFM overexpression inhibited it. These results might help unveil the putative mechanisms or pathways of ASFV replication and its pathogenic effects. Furthermore, the information on host proteins and pathways targeted by ASFV D1133L might contribute to developing novel therapeutic targets against ASFV.

## Data availability statement

The original contributions presented in the study are included in the article/Supplementary material, further inquiries can be directed to the corresponding authors. The mass spectrometry proteomics data presented in the study are deposited in the ProteomeXchange repository, accession number PXD037029.

## Author contributions

YH: conceptualization. YH, JY, and BY: formal analysis. KZ and HZ: funding acquisition. YH, TZ, and XS: investigation. KZ: supervision. YH, XY, DaZ, DeZ, WY, and LC: validation. YH and XL: writing—original draft. KZ, HZ, and BY: writing—review and editing. All authors contributed to the article and approved the submitted version.

## Funding

This work was supported by grants from Major Science and Technology Project of Gansu Province, China (21ZD3NA001-5). This work was also supported by the National Key R&D Program of China (2021YFD1801300), the Major Science and Technology Project of Gansu Province, China (20ZD7NA006-2) and research funding from Lanzhou Veterinary Research Institute, Lanzhou, China (CAAS-ZARW202006-03).

## Conflict of interest

The authors declare that the research was conducted in the absence of any commercial or financial relationships that could be construed as a potential conflict of interest.

The handling editor declared a shared affiliation with the authors at the time of review.

## Publisher’s note

All claims expressed in this article are solely those of the authors and do not necessarily represent those of their affiliated organizations, or those of the publisher, the editors and the reviewers. Any product that may be evaluated in this article, or claim that may be made by its manufacturer, is not guaranteed or endorsed by the publisher.

## Supplementary material

Supplementary Table S1 ASFV D1133L-interacting host proteins.
